# Two-dimensional square lattice polonium stabilized by the spin–orbit coupling

**DOI:** 10.1038/s41598-020-68877-4

**Published:** 2020-07-16

**Authors:** Shota Ono

**Affiliations:** Department of Electrical, Electronic and Computer Engineering, Gifu University, Gifu, 501-1193 Japan

**Keywords:** Materials science, Condensed-matter physics, Theory and computation, Physics, Condensed-matter physics

## Abstract

Polonium is known as the only simple metal that has the simple cubic (SC) lattice in three dimension. There is a debate about whether the stabilized SC structure is attributed to the scalar relativistic effect or the spin–orbit coupling (SOC). Here, we study another phase, two-dimensional (2D) polonium (poloniumene), by performing density-functional theory calculations. We show that the 2D polonium has the square lattice structure as its ground state and demonstrate that the SOC (beyond the scalar relativistic approximation) suppresses the Peierls instability and is necessary to obtain no imaginary phonon frequencies over the Brillouin zone.

## Introduction

Since the discovery of polonium (Po) by Marie and Pierre Curie in 1898, many researches have been made about the physical and chemical properties. The intriguing property of Po is that it has the simple cubic (SC) lattice, $\alpha$-Po phase, as its ground state, quite different from the fact that most elementary metals have face-centered cubic, body-centered cubic, and hexagonal closed packed lattices. Since Po shows a strong radioactivity and is therefore dangerous to humans, theoretical and computational studies are of importance in understanding the origin of the stabilized SC phase. The density-functional theory (DFT) studies have revealed that the scalar relativistic or spin–orbit coupling (SOC) terms are responsible for the stabilization of the SC phase^1–7^. For example, Legut et al. have shown that the scalar relativistic effect is enough for SC Po to be stabilized^2^, whereas Min et al. have shown that the SOC is more important to prevent the phonon softening from taking place^1, 7^. In this paper, we investigate the two-dimensional (2D) case in order to study how the SOC affects on the lattice stability.

The square (SQ) lattice in two-dimension is an analog of the SC lattice in three-dimension. Recently, Nevalaita and Koskinen have performed systematic DFT calculations of 2D metals up to the atomic number of $Z=83$, i.e., bismuth^8^. However, they have shown that most metals are energetically stable in the hexagonal and honeycomb lattices, but unstable in the SQ lattice geometry. An exception is the SQ lattice of ruthenium, which is more stable than the hexagonal and honeycomb lattices by 0.04 eV and 0.38 eV per atom, respectively, while the former energy is comparable to the thermal energy.

In this paper, we study the 2D Po, *poloniumene*, by performing DFT calculations and demonstrate that the poloniumene has the SQ lattice as its ground state. We show that the SOC plays a crucial role in obtaining the phonon band structure with no imaginary phonon frequencies over the Brillouin zone (BZ). By calculating the susceptibility and the 2D Fermi surface with and without the SOC, we show that the SOC suppresses the Peierls instability, giving a dynamical stability to the SQ lattice of poloniumene.

It has been known that the SOC is important for an accurate description of the phonon band structure of heavy metals, such as three-dimensional (3D)^9^ and 2D bismuth (bismuthene)^10^. It is interesting to note that the dynamical stability of metastable platinum with hexagonal closed packed lattice is endowed with SOC^11^. In the present work, we also emphasize that the SOC is mandatory for obtaining no imaginary frequencies in poloniumene. The present study will improve the fundamental understanding of the SOC effect on the lattice dynamics of heavy metals.

The SQ lattice structure is quite rare in two-dimension. Zhao et al. have reported a synthesis of 2D iron having a SQ lattice in graphene pores^12^. However, Shao et al. have shown that such a structure is unstable by using DFT^13^. Recently, Kano et al. have reported that on graphene 2D copper oxide has a SQ lattice^14^, while this is stabilized by the presence of the oxygen atom located at the center of the unit cell. The poloniumene proposed in the present work serves as a valuable example with a rare structure and can have a strong impact in the field of 2D materials.

## Results and discussion

Below we demonstrate how the SQ lattice is stabilized in poloniumene by calculating the total energy, the phonon band structure, and the noninteracting susceptibility as well as the 2D Fermi surface. We show the DFT results based on the generalized gradient approximation (GGA) only, while the same scenario also holds (the SQ lattice is stabilized by SOC) when the local-density approximation (LDA) is used.

### Stable structure

**Figure 1:**
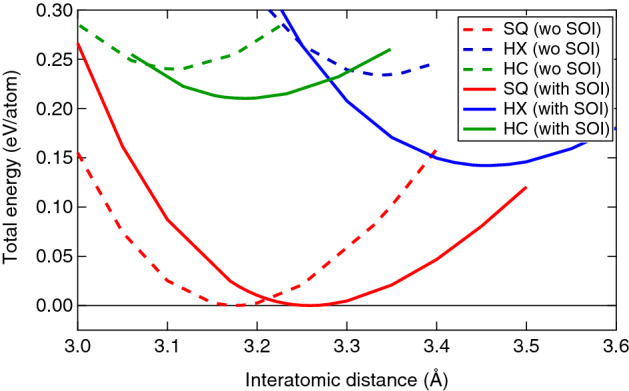
The total energy versus the interatomic distance for SQ, HX, and HC lattices of 2D Po. The total energy is measured from the minimum energy of the SQ lattice structure.

Figure 1 shows the total energy (per atom) as a function of the interatomic bond length (*a*) for three lattice structures: the SQ, hexagonal (HX), and honeycomb (HC) lattices. The curves are calculated without and with the SOC. For both cases, the total energy is measured from that of the most stable geometry of the SQ lattice. The cohesive energy (without SOC) of the SQ lattice is estimated to be 2.58 eV/atom, which is smaller than that of the SC lattice (2.73 eV/atom). The SQ structure is more stable than the HX and HC structures by 0.142 (0.234) eV and 0.210 (0.240) eV with (without) SOC, respectively. These values are larger than the thermal energy of room temperature by an order of magnitude, implying no phase coexistence at ambient conditions. The minimum value of total energy $E_{\mathrm{tot}}$ and the corresponding interatomic distance $a_0$ are listed in Table 1. The size of $a_0$ increases slightly when the SOC is included, similar to Ref.^2^. As the coordination number decreases ($\hbox {HX}\rightarrow \hbox {SQ}\rightarrow \hbox {HC}$), the value of $a_0$ also decreases, so that the lattice constant of the SQ lattice is shorter than that of the SC lattice of 3.359 Å^15^.

**Table 1:** The minimum value of the total energy $E_{\mathrm{tot}}$ (eV/atom) at the equilibrium interatomic distance $a_{0}$ (Å) for SQ, HX, and HC lattices and for the cases without and with the SOC.

	SQ (woSOC)	HX (woSOC)	HC (woSOC)	SQ (wSOC)	HX (wSOC)	HC (wSOC)
$E_{\mathrm{tot}}$	0.0	0.234	0.240	0.0	0.142	0.210
$a_0$	3.175	3.338	3.107	3.258	3.460	3.187

### Phonons

**Figure 2:**
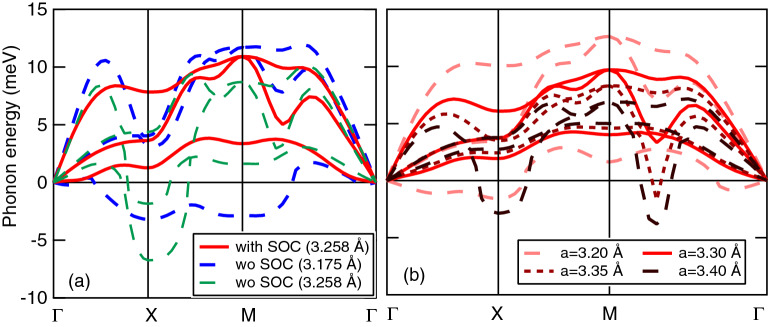
The phonon band structure of poloniumene having the SQ lattice (**a**) with SOC (solid) assuming $a=3.258$ Å and without SOC (dashed) assuming $a=3.175$ and 3.258 Å and (**b**) with SOC assuming $a=3.20, 3.30, 3.35$, and 3.40 Å. The imaginary phonon frequency is expressed by negative phonon energy.

In order to study the dynamical stability of the SQ lattice, we perform the phonon band structure calculations with and without the SOC. Figure 2a shows the dispersion curves along the symmetry lines in the BZ, where the imaginary phonon energy is expressed by negative values. Without the SOC ($a=a_0=3.175$ Å), the imaginary frequencies appear around high symmetry point, X and M, indicating that the SQ lattice structure is unstable. With the SOC ($a=a_0=3.258$ Å), on the other hand, no imaginary frequencies appear. Since the 2D Po without SOC is dynamically unstable when $a=3.258$ Å, i.e., the optimized lattice constant with SOC, the SOC effect is important to stabilize the SQ lattice structure of poloniumene. This scenario is similar to the case of 3D Po, where the SOC plays a key role for the realization of the SC lattice^7^.

We also study how the size of *a* influences the dynamical stability of poloniumene in the presence of SOC. Figure 2b shows the phonon band structure of the 2D Po with $a=3.20, 3.30, 3.35,$ and 3.40 Å. The decrease in *a* from $a=a_0$ yields imaginary frequencies around point X in the lowest phonon branch, while the phonon hardening is observed in the longitudinal acoustic (LA) phonon branch as in the case of 3D Po^7^. The poloniumene is dynamically stable against an elongation of *a*, while it will be unstable when $a\ge 3.35$ Å.

We note some characteristic properties of phonons in poloniumene. Around $\Gamma$, there are three acoustic phonons: the LA, transverse acoustic (TA), and flexural modes. The velocity of the former two branches with a linear dispersion, $v_{\mathrm{L}}$ and $v_{\mathrm{T}}$, is obtained as follows: $v_{\mathrm{L}}^{\mathrm{X}}=3.4$ km/s and $v_{\mathrm{T}}^{\mathrm{X}}=1.0$ km/s along $\Gamma$-X direction and $v_{\mathrm{L}}^{\mathrm{M}}=2.8$ km/s and $v_{\mathrm{T}}^{\mathrm{M}}=2.2$ km/s along $\Gamma$–M direction. These are related to the elastic constants ($c_{11}$, $c_{12}$, and $c_{44}$) as follows: $v_{\mathrm{L}}^{\mathrm{X}}=\sqrt{c_{11}/\rho }$, $v_{\mathrm{T}}^{\mathrm{X}}=\sqrt{c_{44}/\rho }$, $v_{\mathrm{L}}^{\mathrm{M}}=\sqrt{(c_{11}+c_{12}+ 2c_{44})/\rho }$, and $v_{\mathrm{T}}^{\mathrm{M}}=\sqrt{(c_{11}-c_{12})/\rho }$ with $\rho$ the mass density per area^16^. Assuming $\rho = 3.28\times 10^{-6}$ kg/m$^2$, one obtains $c_{11}= 38.7$, $c_{12}\simeq 8.1$, and $c_{44}= 3.3$ GPa nm. The elastic anisotropy of the poloniumene is quite large (i.e., $c_{11}\gg c_{44}$), compared to other 2D metals^8^, but is consistent with the case of 3D Po having the SC lattice^2^.

The origin of the anisotropy of the elastic constants obtained is an intrinsic property of the SQ lattice. To show it qualitatively, we consider a central potential, *V*(*R*), with *R* the interatomic distance up to the second nearest-neighbour (NN) atoms. By diagonalizing the dynamical matrix^17^, one obtains $v_{\mathrm{L}}^{\mathrm{X}} = a_{0} \sqrt{(\xi _1 + \xi _2 + \eta _2)/M}$ and $v_{\mathrm{T}}^{\mathrm{X}} = a_{0} \sqrt{(\xi _2 + \eta _2)/M}$, where *M* is the mass of Po atom, $\xi _l = V''(R_l)$, and $\eta _l = V'(R_l)/R_l$ for $l=1,2$. The derivative (prime) of *V* with respect to *R* is evaluated at $R_l$ the distance of the *l*th NN atoms, i.e., $R_1=a_0$ and $R_2=\sqrt{2}a_0$. Notice that the expression of $v_{\mathrm{T}}^{\mathrm{X}}$ vanishes when $\xi _2$ and $\eta _2$ are neglected. Since the contribution from $l=2$ is generally smaller than that from $l=1$, the inequality $v_{\mathrm{L}}^{\mathrm{X}} \gg v_{\mathrm{T}}^{\mathrm{X}}$ is obtained. A similar discussion holds for the phonon energy at the point N for body-centered cubic lattice^18^.

### Electrons

**Figure 3:**
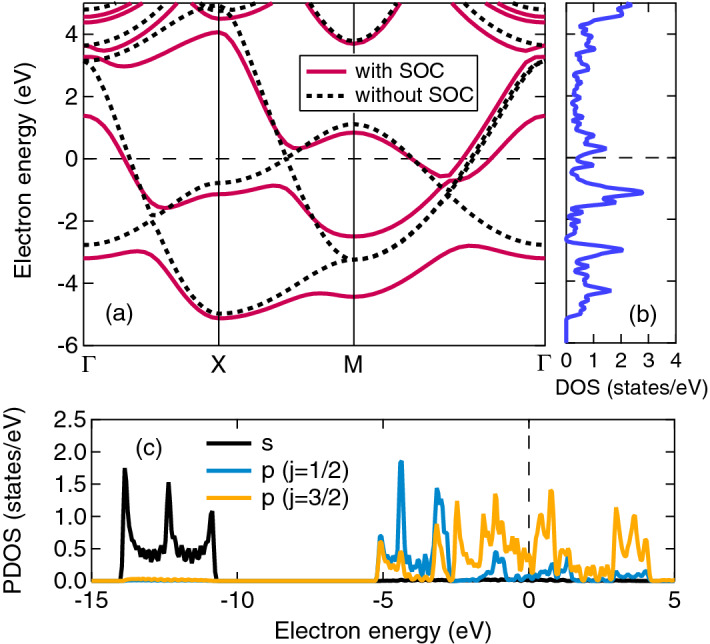
(**a**) The electron band structure of the SQ lattice of poloniumene with and without the SOC. (**b**) The electron DOS around the Fermi level and (**c**) the PDOS that consists of 6*s* and 6*p* electron contributions. The electron energy is measured from the Fermi level.

Figure 3a shows the electron band structure of poloniumene having the SQ lattice, for the cases with (solid) and without (dashed) the SOC. The electron density-of-states (DOS) for the case of with SOC is also shown in Fig. 3b. Without SOC, the band crossings occur at the middle of the lines of $\Gamma$–X, X–M, and M–$\Gamma$. At each point, the band repulsion occurs when the SOC is added, and hence the Fermi level crosses the dispersive band along the lines of $\Gamma$–X and M–$\Gamma$ only. In order to investigate which orbital contributes to the band formation around the Fermi level, we show the projected DOS (PDOS) in Fig. 3c. The 6*p*-states with the total angular momentum $j=3\hbar /2$ ($\hbar$ the Planck constant) are mainly responsible for the formation of the dispersive band. The 6*s* band is located well below the Fermi level by more than 10 eV and decoupled from the 6*p* bands.

### Peierls instability

**Figure 4:**
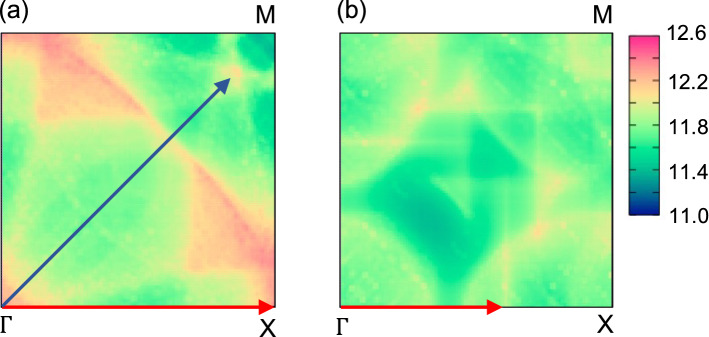
$\chi (\mathbf {q})$ (states/eV) for poloniumene: (**a**) without and (**b**) with the SOC. Examples of the nesting vectors are indicated by arrows: $\mathbf {q}=\mathrm{X}$ and 0.87M for (**a**) and $\mathbf {q}=0.6\mathrm{X}$ for (**b**).

To understand the origin of the dynamical stability of poloniumene, we calculate the noninteracting susceptibility $\chi (\mathbf {q})$ (the wavevector $\mathbf {q}$), provided in “Methods” section, by using the single-particle energy obtained from DFT calculations. Figure 4a,b show $\chi (\mathbf {q})$ for poloniumene without and with the SOC, respectively. Without the SOC, the value of $\chi (\mathbf {q})$ is strongly enhanced around $\mathbf {q}=\mathrm{X}$ and 0.87M, at which in Fig. 2a the phonon softening and imaginary frequencies are observed. With the SOC, on the other hand, such a peak in $\chi (\mathbf {q})$ is smeared out, so that positive phonon energies are obtained in Fig. 2a.

**Figure 5:**
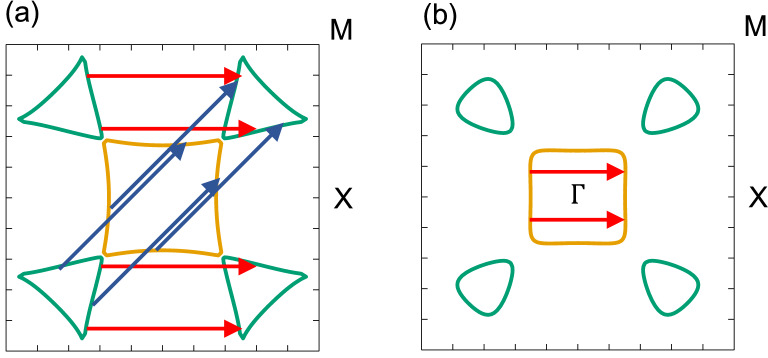
The Fermi line for poloniumene: (**a**) without and (**b**) with the SOC. The center corresponds to the point $\Gamma$. The nesting vectors indicated correspond to those in Fig. 4.

The $\mathbf {q}$-dependence of $\chi$ can be understood in terms of the Fermi surface nesting in two-dimension. Figure 5a,b show the Fermi lines without and with the SOC, respectively. The almost square hole pocket around $\Gamma$ as well as the electron pockets located along the $\Gamma$–M direction are shrinked when the SOC is included. Accordingly, it is difficult to find the nesting vectors on the Fermi lines. In this way, the SOC suppresses the Peierls instability in poloniumene having the SQ lattice.

**Figure 6:**
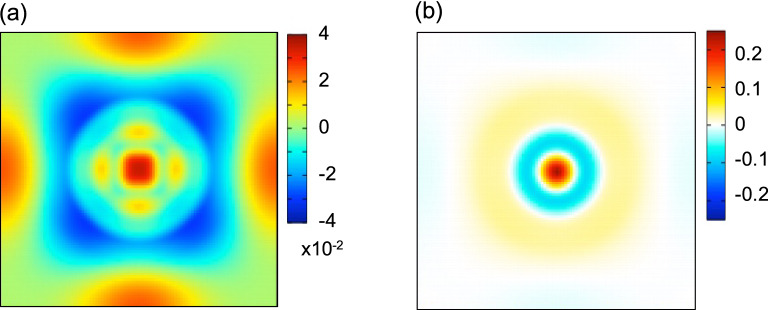
The total charge density (Å$^{-3}$) with the SOC in a unit cell, subtracted by (**a**) the superposition of atomic density and (**b**) the total charge density without the SOC ($a=3.258$ Å is assumed).

It would be helpful to discuss the Peierls instability in view of the charge density analysis. Figure 6a shows the contour plot of the charge density minus the superposition of atomic densities with the SOC, where the Po atom is located at the center of the unit cell. The bonding between the NN sites is strongly enhanced and the SQ lattice symmetry is clearly observed because four 6*p* electrons are occupied below the Fermi level. Figure 6b shows the charge density with the SOC minus that without the SOC, where the size of *a* is fixed to 3.258 Å for both cases to study the impact of SOC only. The effect of the SOC enhances the electron density at the nucleus by 0.244 $e^{-}/$Å$^{3}$, but lowers that at the middle of the NN sites by 0.007 $e^{-}/$Å$^{3}$. The weakening of the directional bonding between Po atoms prevents the poloniumene from the Peierls instability. Similar scenario holds for explaining the origin of the stabilized SC structure of Po in three dimension^1, 7^.

It has been known that the Peierls instability is prone to occur in low dimensional systems^19^. This is because the value of $\chi (\mathbf {q})$ is easily enhanced for low dimension when the nesting vectors are present at the Fermi surface. The Peierls instability has been addressed to occur in 3D Po^1, 5, 7^ because many phonon soft modes are observed in the DFT calculations without SOC, whereas such modes are hardened with SOC included. The debate on the origin of the stabilized SC phase in Po may be replaced with a question how the Peierls instability is suppressed enough to yield no imaginary phonon frequencies. We have demonstrated that in 2D Po the Peierls instability is suppressed not by the scalar relativistic terms but by SOC.

## Methods

To perform DFT calculations, we use the plane-wave based program of Quantum ESPRESSO^20^ and use the projector augmented wave (PAW) pseudopotentials obtained from pslibrary^21^. The valence configuration of Po is $(5d)^{10}(6s)^2(6p)^4$. The effects of exchange and correlation are treated within both LDA^22^ and GGA^23^. The effect of SOC is treated within the scheme described in Refs.^24, 25^. The cutoff energy for the wavefunction is set to be 1360 eV and 1088 eV with and without SOC, respectively. The size of the cutoff energy for the charge density is increased to tenfold that for the wavefunction. The self-consistent field (scf) calculations are performed by using $30\times 30 \times 1 \,k$ Monkhorst-Pack grid^26^, the smearing parameter of 0.34 eV is used, and the vacuum region between the layers is set to be 14 Å. The total energy is converged within $1.36\times 10^{-4}$ eV in the geometry optimization. The phonon band structure calculations are performed based on the density-functional perturbation theory^27^ implemented in Quantum ESPRESSO^20^ and by using $4\times 4\times 1$, $6\times 6\times 1$, and $8\times 8 \times 1 \,q$ grids. We have confirmed that at least $8\times 8 \times 1 \,q$ grid is necessary to obtain no imaginary frequencies over the BZ for the calculations with SOC.

To investigate the Peierls instability of the poloniumene, we calculate the noninteracting susceptibility at the wavevector $\mathbf {q}$

1 $$\begin{aligned} \chi (\mathbf {q}) = - \frac{1}{N_{\mathrm{c}}}\sum _{n,n'}\sum _\mathbf{{k}} \frac{f(\varepsilon _{n'\mathbf {k}+\mathbf {q}}) - f(\varepsilon _{n\mathbf {k}}) }{\varepsilon _{n'\mathbf {k}+\mathbf {q}} - \varepsilon _{n\mathbf {k}}}, \end{aligned}$$

where the matrix elements are neglected^19^. $\varepsilon _{n\mathbf {k}}$ is the electron energy for the wavevector $\mathbf {k}$ and the band index *n*. For the case without SOC, the spin index is included to *n*. $f(\varepsilon )$ is the Fermi distribution function at zero temperature for the electron energy $\varepsilon$. $N_{\mathrm{c}}$ is the number of unit cell. For the summation with respect to $\mathbf {k}$ in Eq. (1), a $100\times 100\times 1 \,k$ grid is used.

## References

[1] Min BI (2006). Origin of the stabilized simple-cubic structure in polonium: Spin–orbit interaction versus peierls instability. Phys. Rev. B.

[2] Legut D, Friák M, Šob M (2007). Why is polonium simple cubic and so highly anisotropic?. Phys. Rev. Lett..

[3] Kim K, Choi HC, Min BI (2009). Comment on "why is polonium simple cubic and so highly anisotropic?". Phys. Rev. Lett..

[4] Šob M, Legut D, Friák M (2009). Šob, legut, and friák reply. Phys. Rev. Lett..

[5] Verstraete MJ (2010). Phases of polonium via density functional theory. Phys. Rev. Lett..

[6] Belabbes A, Zaoui A, Ferhat M (2010). Strong phonon anomalies and fermi surface nesting of simple cubic polonium. Solid State Commun..

[7] Kang C-J, Kim K, Min BI (2012). Phonon softening and superconductivity triggered by spin–orbit coupling in simple-cubic $$\alpha $$-polonium crystals. Phys. Rev. B.

[8] Nevalaita J, Koskinen P (2018). Atlas for the properties of elemental two-dimensional metals. Phys. Rev. B.

[9] Díaz-Sánchez LE, Romero AH, Gonze X (2007). Phonon band structure and interatomic force constants for bismuth: Crucial role of spin–orbit interaction. Phys. Rev. B.

[10] Aktürk E, Aktürk OU, Ciraci S (2016). Single and bilayer bismuthene: Stability at high temperature and mechanical and electronic properties. Phys. Rev. B.

[11] Schönecker S, Li X, Richter M, Vitos L (2018). Lattice dynamics and metastability of FCC metals in the HCP structure and the crucial role of spin–orbit coupling in platinum. Phys. Rev. B.

[12] Zhao J (2014). Free-standing single-atom-thick iron membranes suspended in graphene pores. Science.

[13] Shao Y, Pang R, Shi X (2015). Stability of two-dimensional iron carbides suspended across graphene pores: First-principles particle swarm optimization. J. Phys. Chem. C.

[14] Kano E (2017). One-atom-thick 2d copper oxide clusters on graphene. Nanoscale.

[15] DeSando R, Lange R (1966). The structures of polonium and its compounds-i $$\alpha $$ and $$\beta $$ polonium metal. J. Inorg. Nucl. Chem..

[16] Kittel C (2005). Introduction to Solid State Physics.

[17] Ashcroft N, Mermin ND, Wei D (2016). Solid State Physics.

[18] Ono S (2019). Lattice dynamics for isochorically heated metals: A model study. J. Appl. Phys..

[19] Gr̈uner G (1994). Density Waves in Solids.

[20] Giannozzi P (2017). Advanced capabilities for materials modelling with quantum ESPRESSO. J. Phys. Condens. Matter.

[21] Dal Corso A (2014). Pseudopotentials periodic table: From H to PU. Comput. Mater. Sci..

[22] Perdew JP, Zunger A (1981). Self-interaction correction to density-functional approximations for many-electron systems. Phys. Rev. B.

[23] Perdew JP, Burke K, Ernzerhof M (1996). Generalized gradient approximation made simple. Phys. Rev. Lett..

[24] Dal Corso A (2010). Projector augmented-wave method: Application to relativistic spin-density functional theory. Phys. Rev. B.

[25] Dal Corso A (2012). Projector augmented wave method with spin–orbit coupling: Applications to simple solids and zincblende-type semiconductors. Phys. Rev. B.

[26] Monkhorst HJ, Pack JD (1976). Special points for brillouin-zone integrations. Phys. Rev. B.

[27] Baroni S, de Gironcoli S, Dal Corso A, Giannozzi P (2001). Phonons and related crystal properties from density-functional perturbation theory. Rev. Mod. Phys..

